# Two phytotoxic azaphilone derivatives from *Chaetomium globosum*, a fungal endophyte isolated from *Amaranthus viridis* leaves

**DOI:** 10.1080/21501203.2015.1089332

**Published:** 2015-09-22

**Authors:** K.G. Nelum P. Piyasena, W.A.R.T. Wickramarachchi, N. Savitri Kumar, Lalith Jayasinghe, Yoshinori Fujimoto

**Affiliations:** a Institute of Fundamental Studies, Hantana Road, Kandy, Sri Lanka; b Horticultural Crop Research and Development Institute, Gannoruwa, Peradeniya, Sri Lanka; c Department of Chemistry and Materials Science, Tokyo Institute of Technology, Meguro, Tokyo, Japan

**Keywords:** *Chaetomium globosum*, *Amaranthus viridis*, endophytic fungi, chaetomugilin D, chaetomugilin J, phytotoxcity

## Abstract

A fungal endophyte isolated from the leaves of the allelopathic plant *Amaranthus viridis* of the family Amaranthaceae was identified as *Chaetomium globosum* through molecular means using internal transcribed spacer (ITS) region of rDNA gene. This is the first report of the isolation of *C. globosum* from Amaranthaceae. Chromatographic separation of the EtOAc extract of the fungal fermentation in potato dextrose broth yielded two known chlorine-containing azaphilone derivatives, chaetomugilin D (**1**) and chaetomugilin J (**2**). Compounds **1** and **2** were found to show phytotoxic activity in the lettuce (*Lactuca sativa*) seed germination bioassay. The IC_50_ values of **1** and **2** for root growth inhibition were 24.2 and 22.6 ppm, respectively, while IC_50_ values for shoot growth inhibition were 27.8 and 21.9 ppm, respectively. Phytotoxic activities of the chaetomugilin group of compounds have been reported for the first time, although their antifungal, antimicrobial and cytotoxic activities are known.

## 1. Introduction

The term endophyte refers to a bacterium or a fungus that inhabits living internal organs of higher plants without causing visible pathogenic effect on its host (Strobel and Strobel 2007). Endophytes can be found in virtually all terrestrial plants and may play important role for the growth of hosts (Saikkonen et al. 2004). Endophytic fungi occupy millions of unique biological habitats that are subjected to different environmental interactions and are considered to be a source of rare and important bioactive plant metabolites which may be suitable for medical and for agricultural applications. A range of secondary metabolites, bio-synthesized via various metabolic pathways, have been isolated from endophytic fungi and these metabolites show a broad spectrum of biological activities (Strobel 2002, 2004; Strobel et al. 2004). In a continuation of our studies towards the search for bioactive compounds from Sri Lankan flora, we studied the chemistry and bioactivity of secondary metabolites produced by endophytic fungi from *Amaranthus viridis* (Amaranthaceae). *Amaranthusviridis* is an allelopathic plant that grows in tropical and subtropical regions of the world and is eaten traditionally as a vegetable. The plant is used to treat for liver diseases, gonorrhea, haemorrhoids, snake bite, inflammations, etc. (Jayaweera 1980). Here we report the isolation and identification of an endophytic fungus from the leaves of *A. viridis* as *Chaetomium globosum* and the isolation and characterization of two known azaphilone derivatives, cheatomugilin D (**1**) and cheatomugilin J (**2**) from the fungal fermentation. Phytotoxic activity of compounds **1** and **2** against lettuce seed (*Lactuca sativa*) germination is also described.

## 2. Materials and methods

### 2.1 General

Extractions were taken using a sonicator (VWR Ultrasound cleaner, model-USC 1700 D, Lutterworth, UK). Thin-layer chromatography (TLC) analysis was conducted on silica gel plates (Merck 1.05554.0007, 60F_254_; Darmstadt, Germany). TLC spots were located using a UV lamp and by heating after spraying with acidic anisaldehyde. Silica gel (Merck Art. 7734 and 9385; Darmstadt, Germany) and Sephadex LH-20 (Fluka, Switzerland) were used for column chromatography. ^1^HNMR and ^13^C NMR were recorded on a Bruker DRX500 (Billerica, MA, USA) (500 MHz for ^1^H and 125 MHz for ^13^C) spectrometer in CDCl_3_ solution. Fast atom bombardment mass spectra (FABMS) (+) were obtained on a JEOL JMS-700 spectrometer with 3-nitrobenzyl alcohol as matrix.

### 2.2 Isolation of endophytic fungus

Young healthy leaves of *A*. *viridis* were collected from the Central Province of Sri Lanka in November 2013. Leaves were rinsed with running water and sterilized with ethanol, 5% NaOCl and distilled water three times. A segment of the leaf was placed on potato dextrose agar (PDA) media in a Petri dish (90 mm) and incubated at room temperature. Emerging fungi were isolated after 4 days and sub-cultured to obtain a pure culture of endophytic fungus. The fungus was identified as *C*. *globosum* on the basis of the sequence of internal transcribed spacer (ITS) regions of the fungal rDNA gene, which was amplified using ITS1 and ITS4 primers. BLAST search indicated that the sequence matched 94% with those of six *C. globosum* strains (GenBank accession Nos. HE962482, JX280796, JN209871, HQ914899.1, HQ914911 and GU244529). Photographic evidence of the leaves of *A. viridis* and fungal strain were deposited at the Institute of Fundamental Studies.

### 2.3 Fermentation of the fungus and isolation of secondary metabolites

Large-scale culturing of the fungus was carried out by inoculating *C. globosum* culture grown on PDA medium to Erlenmeyer flasks (1 L × 16), each containing 250 ml of potato dextrose broth (PDB), which was allowed to stand at room temperature for 4 weeks. The resulting culture broth was filtered, and the filtrate and mycelium were separately extracted with EtOAc three times. TLC analysis indicated that the two EtOAc extracts showed a similar TLC pattern. Hence, the two extracts were combined (5.4 g) and screened for phytotoxic activity (Baratelli et al. 2012). Chromatographic separation of the combined EtOAc extracts was by CC using normal-phase silica (Merck 7734) eluted with CH_2_Cl_2_–MeOH gradient. The fractions eluted with CH_2_Cl_2_–MeOH (30:1) were further purified by preparative TLC (PTLC) and Sephadex LH-20 eluted with MeOH and led to the isolation of **1** (6 mg, Qin et al. 2009), **2** (12 mg, Muroga et al. 2009) and ergosterol (6 mg).

*Chaetomugilin D* (**1**): Yellow amorphous solid; ^1^HNMR (CDCl_3_, 500 MHz): *δ* 7.27 (1H, *s*, H-1), 6.55 (1H, *s*, H-4), 2.98 (1H, *d*, 10.1, H-8), 6.06 (1H, *d*, 15.8, H-9), 6.52 (1H, *dd*, 15.8, 8.2, H-10), 2.26 (1H, *m*, H-11), 1.42 (2H, *m*, H-12), 0.90 (3 H, *t*, 7.4, H-13), 1.41 (3H, *s*, 7-Me), 1.08 (3H, *d*, 6.7, 11-Me), 3.03 (1H, *d*, 10.1, H-1ʹ), 1.90 (1 H, *dq*, 10.0, 6.9, H-3ʹ), 4.30 (1H, *dq*, 10.0, 6.4, H-4ʹ), 1.41 (3H, *d*, 6.4, H-5ʹ), 1.13 (3H, *d*, 6.9, 3ʹ-Me), ^13^CNMR (CDCl_3_, 125 MHz): *δ* 145.6 (C-1), 157.7 (C-3), 105.0 (C-4), 140.5 (C-4a), 110.1 (C-5), 189.3 (C-6), 83.9 (C-7), 50.5 (C-8), 114.3 (8a), 120.2 (C-9), 146.9 (C-10), 38.9 (C-11), 29.2 (C-12), 11.7 (C-13), 23.3 (C-7-Me), 19.4 (C-11-Me), 58.3 (C-1ʹ), 104.2 (C-2ʹ), 44.9 (C-3ʹ), 76.9 (C-4ʹ), 18.7 (C-5ʹ), 8.8 (C-3ʹ-Me), 170.6 (C-1”); FABMS *m*/*z* 435 [M + H for ^35^Cl]^+^ and 437 [M + H for ^37^Cl]^+^.

*Chaetomugilin J* (**2**): Yellow amorphous solid; ^1^HNMR (CDCl_3_, 500 MHz): *δδ*7.48 (1H, *s*, H-1), 6.44 (1H, *s*, H-4), 1.33 (3H, *s*, 7-Me), 3.48 (1H, *dd*, 10.6, 2.3, H-8), 6.02 (1H, *d*, 15.5, H-9), 6.48 (1 H, *dd*, 15.7, 8.0, H-10), 2.24 (1H, *sept*, 7.0, H-11), 1.07 (3H, *d*, 6.7, 11-Me), 1.40 (1H, *m*, H-12), 0.88 (3H, *t*, 7.5, H-13), 2.62 (1H, *dd*, 17.7, 10.6, Ha-1ʹ), 3.28 (1H, *dd*, 17.7, 2.4, Hb-1ʹ), 1.73 (3H, *s*, 3ʹ-Me), 6.66 (1H, *q*, 7.1, H-4ʹ), 1.80 (*d*, 7.1, H-5ʹ); ^13^CNMR (CDCl_3_, 125 MHz): *δ* 145.8 (C-1), 157.2 (C-3), 104.5 (C-4), 141.8 (C-4a), 104.6 (C-5), 191.9 (C-6), 74.1 (C-7), 26.8 (C-7-Me), 40.6 (C-8), 119.6 (C-8a), 120.4 (C-9), 146.1 (C-10), 38.8 (C-11), 19.4 (C-11-Me), 29.2 (C-12), 11.7 (C-13), 34.9 (C-1ʹ), 199.5 (C-2ʹ), 138.0 (C-3ʹ), 11.0 (C-3ʹ-Me), 138.1 (C-4ʹ), 14.7 (C-5ʹ); FABMS *m*/*z* 391 [M + H for ^35^Cl]^+^ and 393 [M + H for ^37^Cl]^+^.

### 2.4 Lettuce seed germination bioassay

Seeds of *L*. *sativa* (Rapido 344, East West Seed International Ltd., Nonthaburi, Thailand) were treated with 5% clorox solution for 10 min, rinsed five times with sterile distilled water and kept for 10 min in sterile distilled water. Floating immature seeds were discarded. Five seeds were kept in each Petri dish containing 800 μL of appropriate concentrations of the EtOAc extract (at 1000 ppm), and compound **1** and compound **2** (ranging from 200 to 10 ppm) were dissolved in 1% DMSO in distilled water. Control dishes received 800 μL aliquot of distilled water containing 1% DMSO. After seeds were placed on moistened filter paper, (Whatman No. 4) Petri dishes were incubated in the dark at 25ºC for 5 days. At the end of the incubation period, the number of seeds germinated in each Petri dish was counted, and the length of the root and shoot was measured to the nearest millimetre. Similarly, 800 μL aliquot of abscisic acid (10 ppm solution) was used as the positive control, which exhibited 100% inhibition of lettuce seed germination. In lettuce seed germination bioassay, four replicates were used for each treatment and the experiment was carried out twice (Baratelli et al. 2012).

## 3. Results and discussion

**Figure 1.**
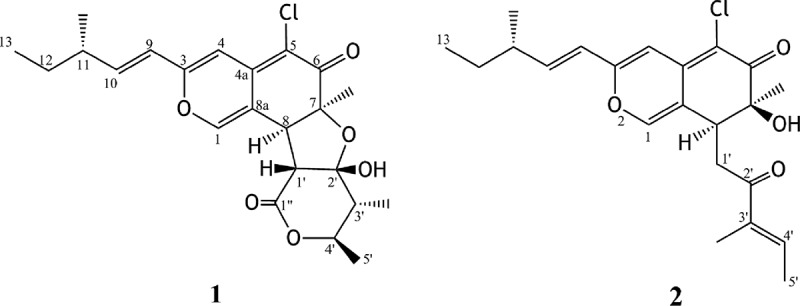
Structure of compounds **1** and **2**.

A fungal endophyte isolated from the leaves of the allelopathic plant *A. viridis* was identified as *C. globosum* on the basis of the sequence of ITS region of rDNA gene. A pure culture of *C. globosum* was fermented in PDB media. The broth and mycelium were separately extracted with EtOAc. Based on the TLC analysis, two EtOAc extracts were combined. The combined EtOAc extract inhibited completely lettuce seed germination at 1000 ppm. Chromatographic separation of the EtOAc extract over silica gel, Sephadex LH-20 and PTLC afforded three compounds, which were identified as chaetomugilin D (**1**) (Qin et al. 2009), chaetomugilin J (**2**) (Muroga et al. 2009) and ergosterol by detailed analysis of ^1^H & ^13^CNMR data and FABMS (Figure 1). The original NMR spectra of compounds **1** and **2** are given in Supplementary material. Compounds **1** and **2** inhibited (100%) lettuce seed germination at 100 ppm. IC_50_ values for percentage root growth inhibition of **1** and **2** were 24.2 and 22.6 ppm, respectively, while IC_50_ values of shoot growth inhibition were 27.8 and 21.9 ppm, respectively.

*Chaetomiumglobosum* is a popular fungus found on damp building materials (McMullin et al. 2013) and was previously isolated as an endophytic fungus from *Ginkgo biloba* (Ginkgoaceae) (Qin et al. 2009), *Viguiera robusta* (Asteraceae) (Borges et al. 2011) and the marine fish *Mugil cephalus* (Yamada et al. 2008). The isolation of chaetomugilin D (**1**) from the fungal endophyte *C. globosum* was reported previously (Muroga et al. 2008; Qin et al. 2009). This is the second report of the isolation of chaetomugilin J (**2**) (Muroga et al. 2009). Chaetomugilin-type compounds are known to have potent cytotoxic (including against brine shrimp larvae), antibacterial and antifungal activities (Muroga et al. 2008, 2009; Qin et al. 2009). Phytotoxic activities of **1** and **2** are reported for the first time in the present study, suggesting a potential use of the two compounds as a herbicide or weedicide to replace or reduce the use of hazardous synthetic compounds.

## Disclosure statement

No potential conflict of interest was reported by the authors.

## Supplementary data

Supplemental data for this article can be accessed at 10.1080/21501203.2015.1089332

Supplementary_material.docx
