# Supplementary material for: Two phytotoxic azaphilone derivatives from Chaetomium globosum, a fungal endophyte isolated from Amaranthus viridis leaves
Source: Mycology. 2015 Sep 22;6(3-4):158–60. doi: 10.1080/21501203.2015.1089332 (PMC6106071; doi:10.1080/21501203.2015.1089332)
Supplement: Supplementary_material.docx [file TMYC_A_1089332_SM6440.docx]

**SUPPLEMENTARY MATERIAL**

**Two phytotoxic azaphilone derivatives from *Chaetomium globosum*, a fungal endophyte isolated from *Amaranthus viridis* leaves**

**Abstract**

A fungal endophyte isolated from the leaves of the allelopathic plant *Amaranthus viridis* of the family Amaranthaceae was identified as *Chaetomium globosum* through molecular means using internal transcribed spacer (ITS) region of rDNA gene. This is the first report of the isolation of *C. globosum* from Amaranthaceae. Chromatographic separation of the AcOEt extract of the fungal fermentation in potato dextrose broth yielded two known chlorine-containing azaphilone derivatives, chaetomugilin D (**1**) and chaetomugilin J (**2**). Compounds **1** and **2** were found to show phytotoxic activity in the lettuce (*Lactuca sativa*) seed germination bioassay. The IC_50_ values for root growth inhibition of **1** and **2** were 24.2 and 22.6 ppm, respectively, while IC_50_ values for shoot growth inhibition were 27.8 and 21.9 ppm, respectively. Phytotoxic activity of the chaetomugilin group of compounds are reported for the first time, although their antifungal, antimicrobial and cytotoxic activities are known.

.

^1^HNMR and ^13^CNMR spectra of compounds**1** and **2** are given below


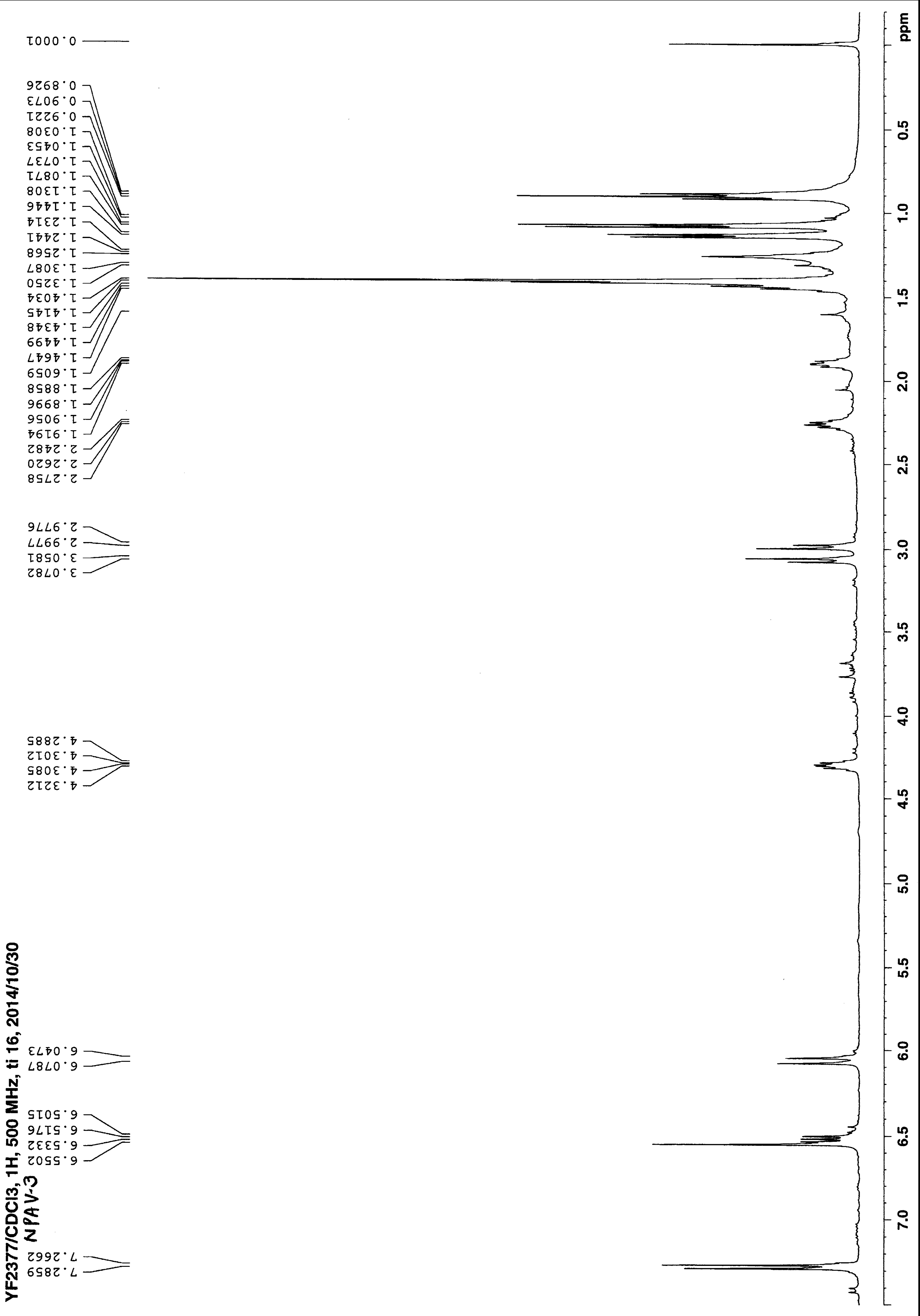


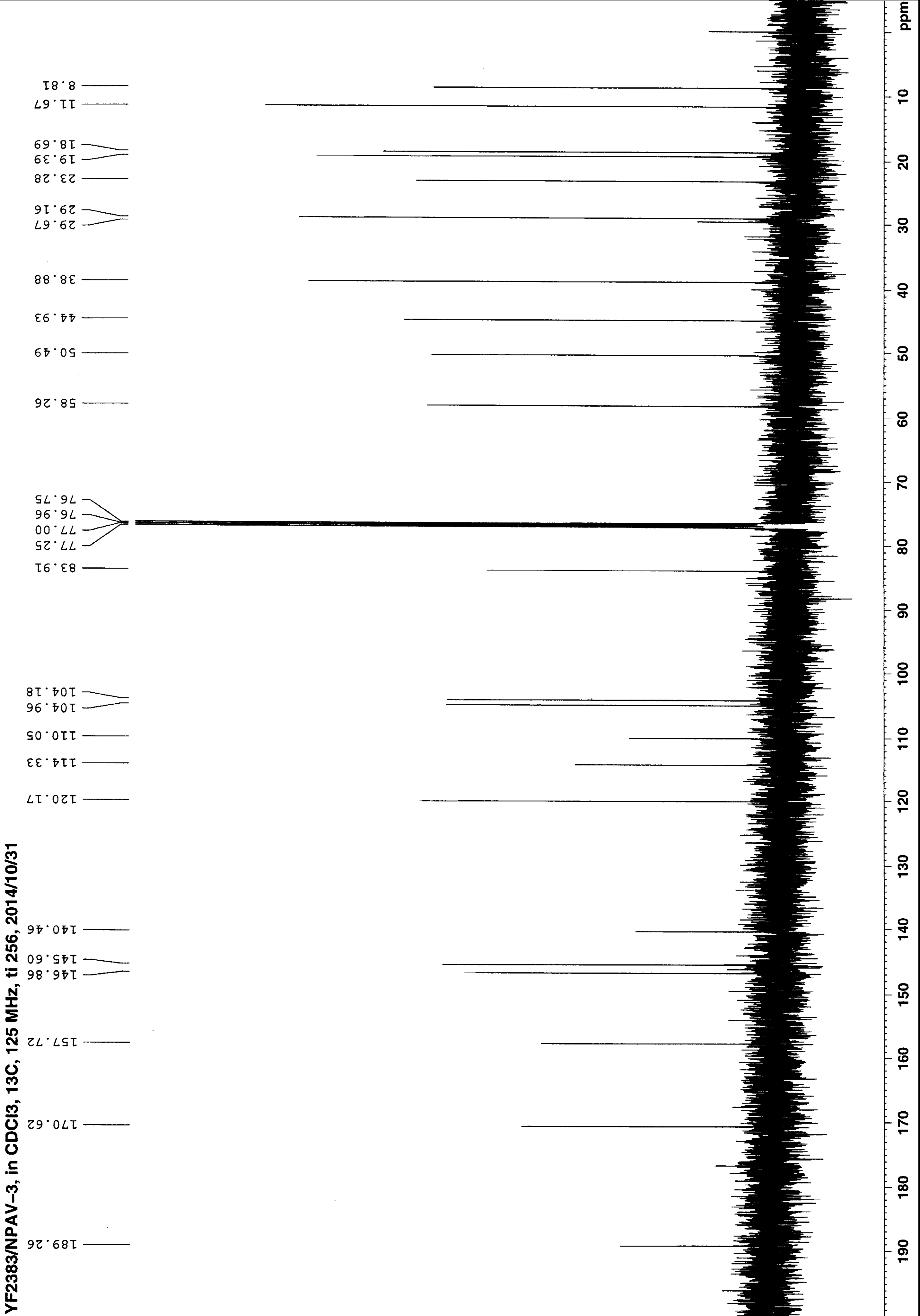


^1^H-NMR spectrum (500 MHz, CDCl_3_) of compound **1**.

^13^C-NMR spectrum (125 MHz, CDCl_3_) of compound **1**.


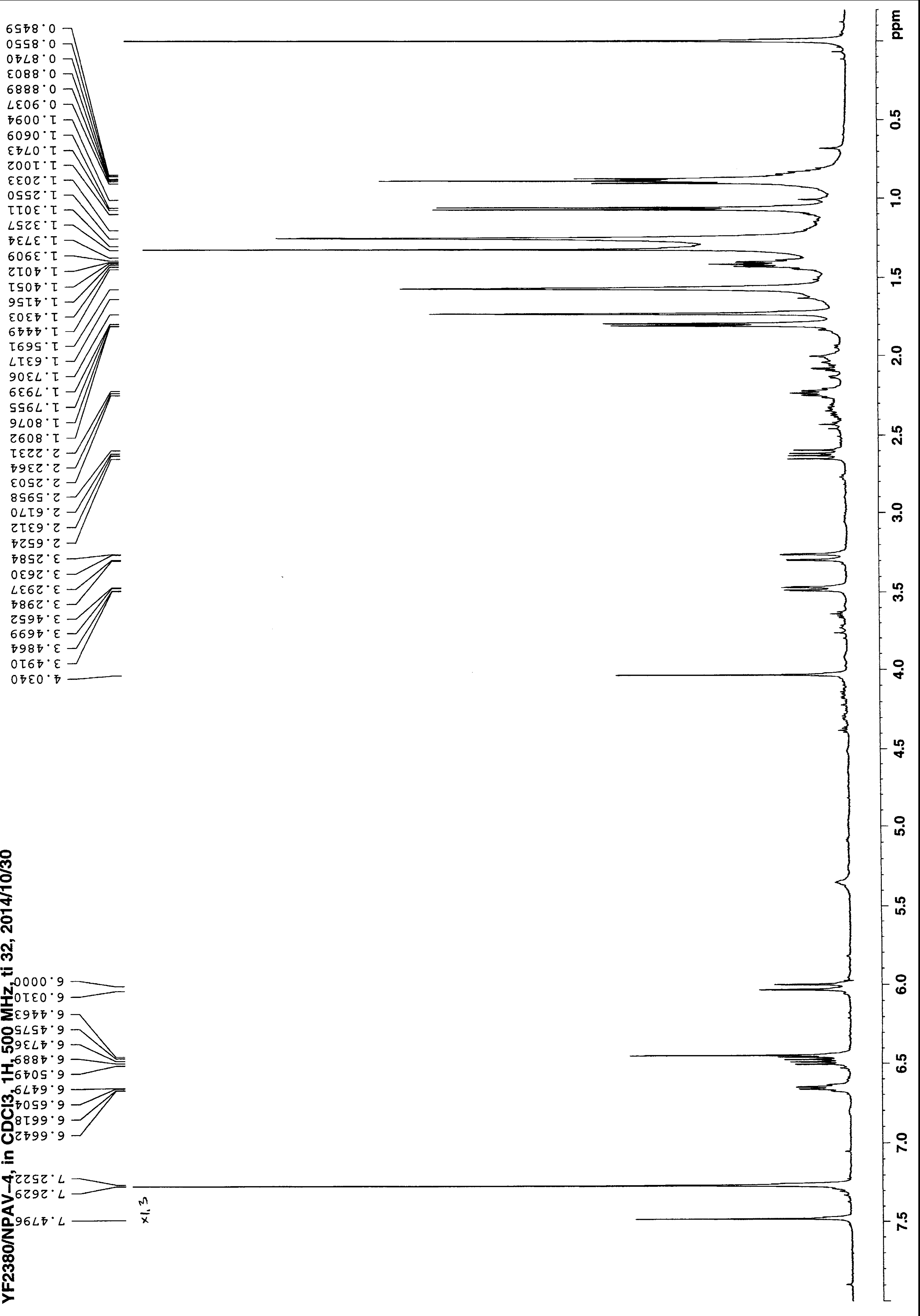


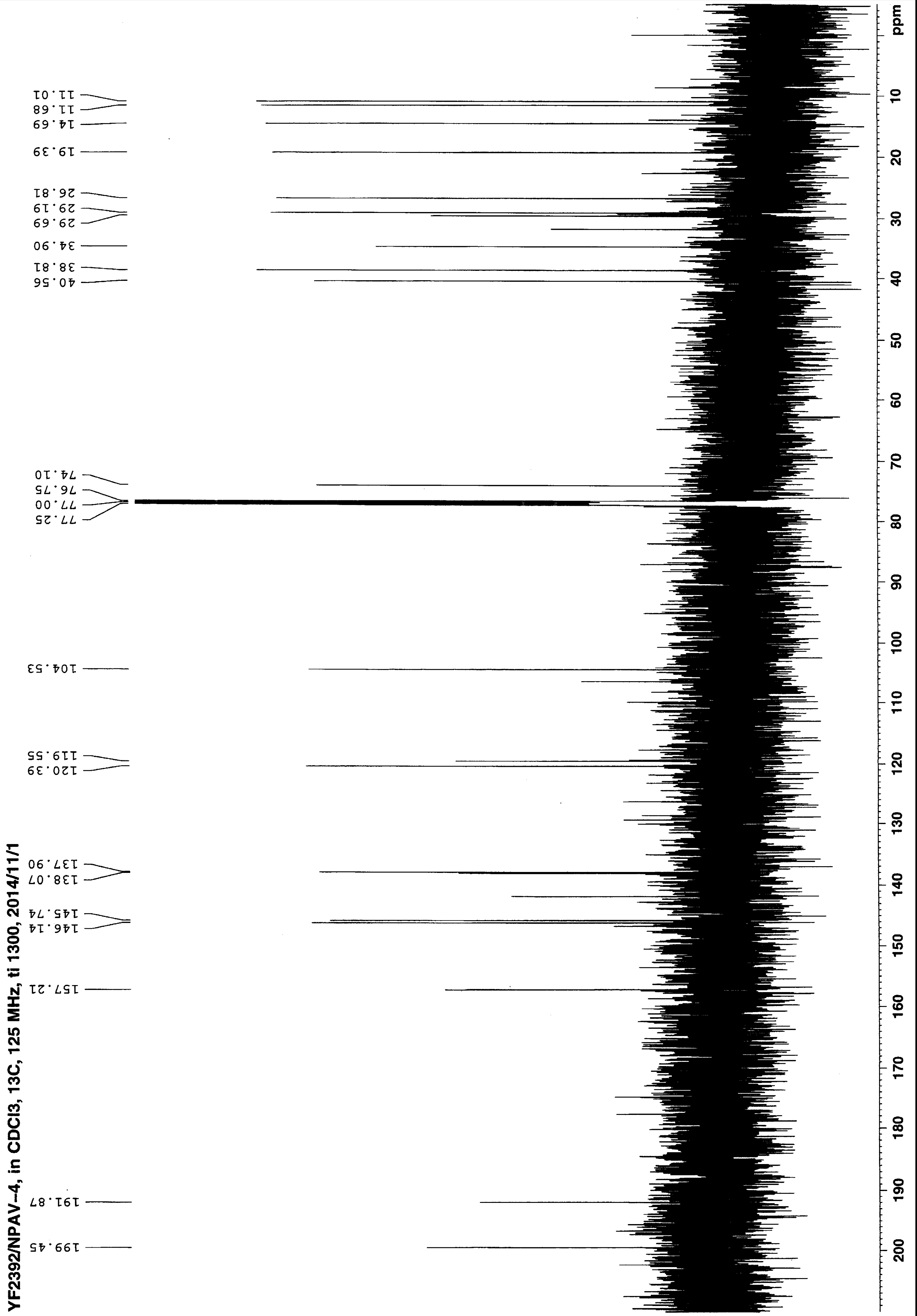


^1^H-NMR spectrum (500 MHz, CDCl_3_) of compound **2**.

^1^H-NMR spectrum (125 MHz, CDCl_3_) of compound **2**.
